# Supplementary material for: Mitochondria-Targeted Catalase Does Not Suppress Development of Cellular Senescence during Aging
Source: Biomedicines. 2024 Feb 10;12(2):414. doi: 10.3390/biomedicines12020414 (PMC10886841; doi:10.3390/biomedicines12020414)
Supplement: Supplementary file 1 [file biomedicines-12-00414-s001.zip › Figure S1.pdf]

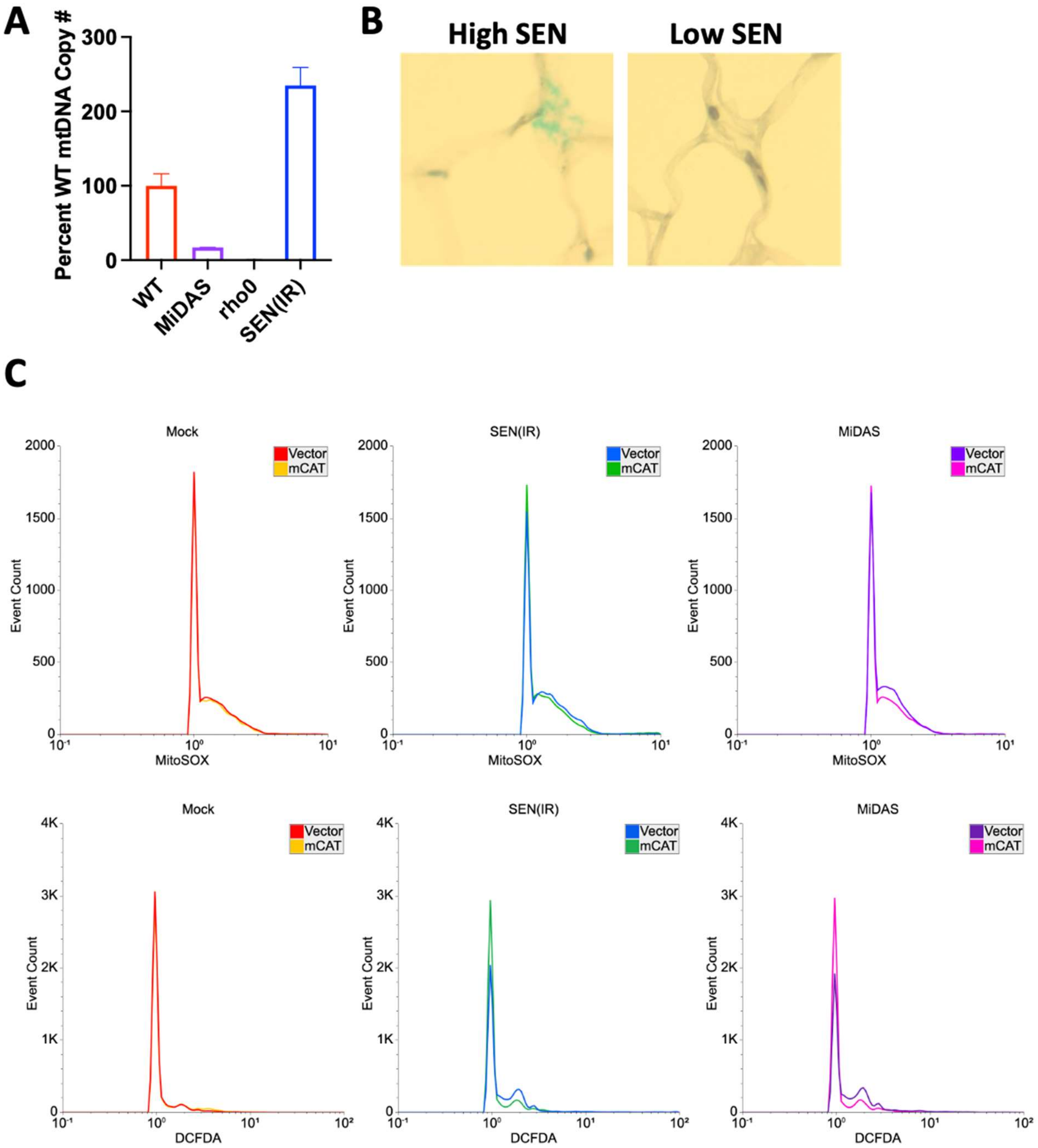

Figure S1. (**A**) Cells from Figure 1 were analyzed for mtDNA levels, normalized to nDNA and expressed as a percentage of WT. (**B**) Example of HMGB1 (IHC, dark nuclei) and senescence-associated beta-galactosidase (blue staining) in high and low senescence adipose tissue images. (**C**) Histograms of vector or mCAT-transduced Mock, SEN(IR), and MiDAS cells stained with MitoSOX or DCFDA (as in Figure 1A,B).
